# Reprogramming: Emerging Strategies to Rejuvenate Aging Cells and Tissues

**DOI:** 10.3390/ijms22083990

**Published:** 2021-04-13

**Authors:** Quentin Alle, Enora Le Borgne, Ollivier Milhavet, Jean-Marc Lemaitre

**Affiliations:** 1 IRMB, University of Montpellier, INSERM, 34295 Montpellier, France; quentin.alle@inserm.fr (Q.A.); enora.le-borgne@inserm.fr (E.L.B.); 2 IRMB, University of Montpellier, INSERM, CNRS, 34295 Montpellier, France

**Keywords:** aging, senescence, epigenetics, stem cells, reprogramming, iPSC

## Abstract

Aging is associated with a progressive and functional decline of all tissues and a striking increase in many “age-related diseases”. Although aging has long been considered an inevitable process, strategies to delay and potentially even reverse the aging process have recently been developed. Here, we review emerging rejuvenation strategies that are based on reprogramming toward pluripotency. Some of these approaches may eventually lead to medical applications to improve healthspan and longevity.

## 1. Introduction

As we age, we become increasingly vulnerable to age-related diseases. The progressive aging of the population makes this issue one of, if not the, major current scientific concern in the field of medicine. Aging is an intricate process that increases the likelihood of cancer, cardiovascular disorders, diabetes, atherosclerosis, neurodegeneration and age-related macular degeneration. The regenerative capacity of cells and tissues diminishes over time and they thus become vulnerable to age-related malfunctions that can precipitate death. Developing prophylactic strategies to increase the duration of healthy life and promote healthy aging is challenging, as the mechanisms causing aging are poorly understood, even if great progress has been made from studying naturally occurring or accelerated-aging phenomena. We now know that aging inculcates many changes, or ‘hallmarks’: genomic instability, telomere shortening, epigenetic alterations, loss of proteostasis, cellular senescence, mitochondrial dysfunction, deregulated nutrient sensing, altered intercellular communication, and stem cell compromise and exhaustion [1].

These various hallmarks of aging are all active fields of molecular mechanistic study with much promise but relatively few tangible results have been translated into therapy.

Perhaps the most effective strategies so far have been those that focus on the removal of senescent cells with ‘senolytic’ drugs [2,3]. In some ways, however, we feel this is too focused on the symptoms of aging whereas perhaps the most promising strategy for the future would be to focus on the causes of aging and its corollary, the rejuvenative capacity of stem cells.

Simply expressing four transcription factors, OCT4, SOX2, KLF4 and c-MYC (OSKM), converts somatic cells into induced pluripotent stem cells (iPSCs) [4]. Reprogramming occurs through a global remodeling of the epigenetic landscape that ultimately reverts the cell to a pluripotent embryonic-like state, with properties similar to embryonic stem cells (ESCs). This cellular reprogramming allows us to imagine cell therapies that restore organ and tissue function. Indeed, by reprogramming a somatic cell, from a donor into iPSCs, these cells can then be modified or corrected before redifferentiation, to produce ‘rejuvenated’ cells, tissues or organs, for replacement in the same donor or an immune-compatible person. In recent years, emerging results have led to new ideas demonstrating that the mechanics of cellular reprogramming can be used to reduce the deleterious effects of aging and to delay these effects by increasing regenerative capacity, either at the cellular or the whole-organism level.

In this review, then, we focus on emerging strategies that aim to rejuvenate cells or tissues based on stem cells, with an emphasis on cell reprogramming approaches that promise new routes for everyone to enjoy prolonged healthspan and lifespan.

## 2. Understanding the Aging Process

Aging brings increasing frailty. There are two major phases during aging. The first phase is healthy aging, where minor alterations accumulate. Then there is a second phase, so-called pathological aging, in which chronic clinical diseases and disabilities predominate and impair physiological functions [5].

The problems facing our aging population can be studied with a new demographic metric, the Healthy Life Years (HLY) or ‘disability-free life expectancy’ [6], which is defined by the European Statistical Office as the average number of years one can expect to live in the absence of these disorders, within the life expectancy and for a given age.

### 2.1. Age-Associated Pathologies

Deterioration of body functions with age is the main risk factor for major human pathologies and therefore the main factor limiting HLY. Moreover, since advanced age is the common causal influence, these chronic disorders often occur concurrently, as comorbidities, in the elderly [1,5]. Among these major pathologies are cancer, most commonly lung, breast, prostate, and colorectal cancers, and cardiovascular disorders including chronic ischemic heart disease, congestive heart failure, and arrhythmia. The latter two heart diseases are now the two leading causes of death [7,8]. Age-related diseases affecting the skeletal system are also common, particularly osteoarthritis and osteoporosis. Another disease that increases greatly with age is the muscular degeneration known as sarcopenia. Metabolic disorders such as diabetes and non-alcoholic hepatic steatosis also become more common with age [9]. Organ and tissue fibrosis, a pathological process characterized by inflammatory injury and excessive fibrous connective tissue production [10], also increases during aging and acts as one of the primary causes for age-related deterioration of human organs, including the lungs [11], kidneys [12], liver [13] and heart [14]. Lymphoid organs, such as the spleen, also undergo a structural loss of integrity in the elderly. Global deterioration of the immune system increases susceptibility to infectious diseases and reduces the response to vaccination [15]. This has been widely illustrated lately by age-related mortality from COVID-19. Finally, there are neurodegenerative diseases, such as Alzheimer’s disease, Parkinson’s, and Huntington’s disease and sensorial failures such as auditory and macular degeneration that all increase significantly in the aged [16,17,18].

The progressive functional and physiological decline of any living organism, leading inevitably to death, is the progressive accumulation of molecular and cellular damage occurring throughout its life.

Thus, aging is not a disease in itself but rather a biological process whose multiple causes and consequences add up and overlap.

### 2.2. Cellular Damage at the Heart of Aging

**Figure 1:**
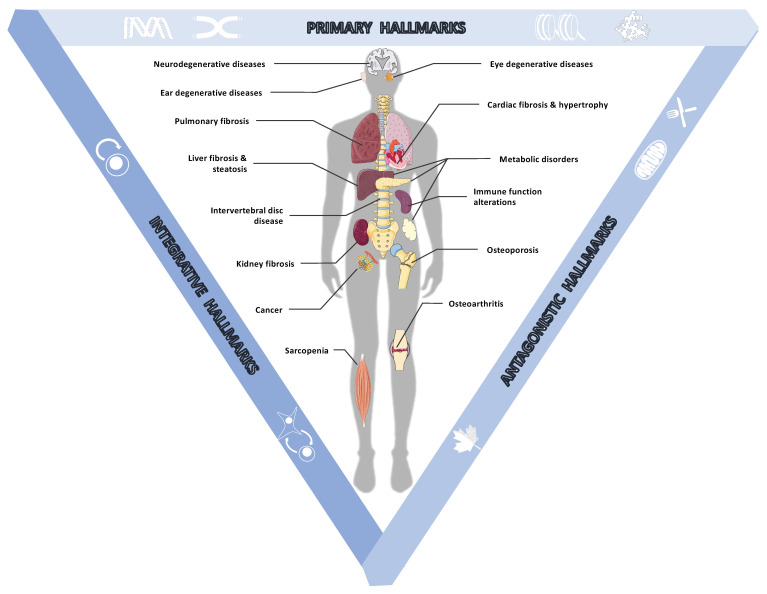
Hallmarks of aging at the origin of age-related diseases. Aging is characterized by a progressive loss of biological functions linked to the appearance and accumulation of molecular and cellular damage over entire lives. This damage has been classified into three categories by López-Otín [1]. (i) Primary hallmarks corresponding to molecular disorders occurring in cells: genomic instability, telomere attrition, epigenetic alterations, and loss of proteostasis. (ii) Antagonistic hallmarks, corresponding to alterations of damage response mechanisms: deregulated nutrient sensing, mitochondrial dysfunction, and cellular senescence. Finally, (iii) integrative hallmarks corresponding to tissue homeostasis failures: stem cell exhaustion and altered intercellular communication. Altogether, these interconnected hallmarks of aging act as cause and catalyst engendering a large set of age-related pathologies affecting the whole body.

For decades, a large number of studies aimed at understanding the adverse effects of aging were carried out on a wide range of model organisms. In 2013, López-Otín et al. compiled much of this knowledge and referenced nine general hallmarks of aging in living organisms [1]. These hallmarks of aging affect the organism at different scales. Some occur at the molecular level within cells, while others impact tissues and even beyond, at the level of an organ or the entire organism. These elements were classified according to three important criteria. First, each hallmark must occur naturally during physiological aging. In addition, the experimental deterioration of each mark must accelerate aging, while, conversely, the experimental improvement of each mark must slow aging. Moreover, as aging occurs, all these hallmarks are gradually implemented and interact with each other and an integrative model of these events was proposed [1] that supports a multifactorial origin of age-related pathologies (Figure 1).

#### 2.2.1. The Primary Hallmarks of Aging Are the Triggering Events Whose Harmful Consequences Progressively Accumulate over Time

The hallmarks are structural changes to biological molecules that alter their functions. These changes increase molecular disorder, or decrease molecular fidelity, within cells. Molecular disorder can be blocked, or at least slowed, by repair and replacement processes. However, these mechanisms are also achieved by biomolecules, which are themselves subject to this increasing disorder [19]. The paradigms of aging-linked disorders are in the macromolecules, DNA, and protein, including genomic instability [20,21,22,23], telomere shortening [24,25,26], epigenetic alterations in DNA [27,28,29], and loss of proteostasis [30,31,32,33,34,35].

#### 2.2.2. The Antagonistic Hallmarks Are Damage Response Mechanisms That Become Overwhelmed

In principle, antagonistic hallmarks of aging are activated to counter the primary hallmarks, but they progressively become negative in a process that is partly favored or accelerated by the primary damage.

Cells suffer many impairments, affecting all their molecules and compartments. Fortunately, they usually have the necessary weapons to deal with these problems. However, as we age, molecular chaos overwhelms our cells’ declining capacity for control and repair. To temporarily stabilize and then eliminate overly damaged cells, we have cellular processes such as senescence. However, senescent cells accumulate within tissues during aging, in particular due to a decrease in their elimination by the immune system, and this accumulation incurs many age-related diseases [36]. Moreover, not only cells but also cell organelles can be damaged. Damaged mitochondria accumulate during aging, upregulating reactive oxygen species and decreasing energy levels and cellular respiratory capacity [37,38,39].

During aging, there is a general deregulation of the nutrient-sensing pathways that detect the intracellular and extracellular levels of nutrients and metabolites as well as the different hormones that regulate them, and several metabolic alterations thus accumulate over time, reducing functionality in metabolic disorders.

In addition, certain environmental factors act as catalysts of these deregulations such as hypercaloric nutrition and a sedentary lifestyle [40].

#### 2.2.3. The Integrative Hallmarks Are Tissue Homeostasis Failures

Integrative hallmarks occur when the accumulated damage caused by the primary and antagonistic hallmarks cannot be compensated for by homeostatic mechanisms within the aging tissues. Indeed, as we age, we witness the gradual accumulation of molecular damage that is no longer tolerated by cellular control mechanisms and thus the number of altered, dysfunctional senescent cells within tissues increases.

Reduced regenerative capacity and/or depletion of stem cells, resulting from accumulated cell damage, are among the major causes of the body aging process [41,42].

These important changes interfere with interactions and communication between cells, tissues, and organs, and result in the loss of tissue integrity. Senescent cells have a specific senescence-associated secretory phenotype (SASP) repertoire composed of pro-inflammatory cytokines (IL-1α, IL-6, IL-8), chemokines (CCL2, CXCL1), growth factors (VEGF), and metalloproteinases (MMP-1, MMP-3). SASP is a major source of circulating inflammatory factors [43,44]. The immune system itself also progressively declines in function over life. This decline, called immuno-senescence, reduces both humoral and cellular immune responses [45,46]. Immuno-senescence also favors a pro-inflammatory environment affecting endocrine, neurocrine, and neuronal intercellular communication.

Although the classification proposed by López-Otín is widely accepted, a few new hallmarks of aging have been identified since 2013, including stiffening of the extracellular matrix [47], tRNA-derived fragments [48], circRNA accumulation [49], and even microbiota dysbiosis [50].

## 3. The Promise of Pluripotent Stem Cells

Among the approaches to age-related pathological phenotypes, most are aimed at preventing or mitigating cell damage [1]. This involves activating cellular stress resistance mechanisms, either with antioxidant molecules or by suppressing senescent cells to reduce their impact on tissues.

An exception is heterochronic parabiosis, which aims at restoring the regenerative ability of older tissues through exposure to circulating juvenile factors [51,52,53,54,55,56,57].

This objective of restoring functions of a tissue or an organ, when the regenerative ability of older tissues is reduced, is a foundation of regenerative medicine.

Thus, new strategies are currently being developed around stem cells and the use of their regenerative potential to prevent the detrimental effects of aging. In particular, human pluripotent stem cells (hPSCs) including ESCs and, more recently, iPSCs, are an indefatigable source of cells for clinical use [58]. ESCs and iPSCs are pluripotent and therefore have the ability to differentiate into any cell type of the body (with the exception of embryonic appendices). This characteristic, in addition to self-renewal, gives hPSCs a central role in a growing number of new cell therapies aimed at restoring functions of many tissues during aging.

### 3.1. Human Embryonic Stem Cells

ESCs were first obtained in mice [59,60] and in rhesus monkeys [61]. The work in primates paved the way for the first successful human embryonic stem cells (hESCs) to be derived a few years later [62]. Characterization of hESCs revealed specific surface markers expressed by these cells, and their ability to differentiate into the three embryonic layers: endoderm, ectoderm, and mesoderm. Following this breakthrough, a large number of studies demonstrated the possibility of differentiating ESCs into different specialized cell types, including mature neurons, cardiomyocytes, or insulin-producing cells [63], thus paving the way for future therapeutic applications.

### 3.2. Cell Reprogramming

Other methods aim to revert to the pluripotent state using somatic cells as starting material. Cellular reprogramming has revolutionized the understanding of many fields of biology and medicine, notably following the discovery of iPSCs in 2006. Two of the main contributors to cell reprogramming were awarded the Nobel Prize in Medicine in 2012, namely, Sir John Gurdon and Shinya Yamanaka [64].

Following the discoveries made in the field of somatic cell reprogramming by nuclear transfer [65,66], which led to therapeutic cloning, trans-differentiation, and cell fusion [67], it has been hypothesized that somatic cells can be directly reprogrammed into pluripotent cells through the action of appropriate transcription factors [68,69,70].

In 2006, Shinya Yamanaka’s team validated this hypothesis with mouse and human cells [4,71]. They determined the minimum cocktail of factors necessary to generate cell colonies similar to those observed in ESC cultures. A final combination of four protein factors, since named Yamanaka factors or OSKM, reprograms somatic cells into induced pluripotent stem cells (iPSCs). OSKM is OCT4 and SOX2, which are stabilizers of pluripotency in ESCs and the early embryo [72,73,74], and KLF4 and C-MYC, which are important in the self-renewal and proliferation of ESCs in culture [75,76]. This discovery revolutionized stem cell research for two main reasons. The first is that this method is completely free of the ethical problems associated with the manipulation of human embryos for research purposes. The second, resulting directly from the first, is that it opens the door to autologous transplant strategies into a much larger space than was possible through classical somatic cell reprogramming by nuclear transfer. With iPSCs, autologous transplants of “reconstructed or repaired” cells, tissues, or organs can be derived from the patient’s own cells, which avoids any risk of rejection down the line. Induced reprogramming represents the third and most recent source of hPSCs developed for therapeutic applications, after therapeutic cloning and deriving ESCs from embryos.

### 3.3. Human Pluripotent Cells as an Experimental Modelling Tool

Reprogramming has revealed that cellular fate is highly plastic. Another parameter of prime importance for medical research is that, after having ascended the slope from one cell type to a pluripotent state, the cell can be brought back down along various different pathways from the original one. Thus, hPSCs create the possibility of in vitro differentiation into various cell types. In vitro differentiation can be used experimentally, to model different diseases, and therapeutically, to manipulate diseased states. In the following sections, we will discuss concrete examples, in the context of aging, of in vitro modelling of differentiation and pathologies, and the challenges of developing them into therapeutic solutions.

#### 3.3.1. Organoids and Complex Tissues

Pluripotent stem cells (PSCs) spontaneously differentiate when culture conditions no longer stabilize their pluripotency. Equally, PSCs can be guided towards desired cell identities if specific stimuli are added, such as those present during embryonic differentiation. Examples of iPSC differentiation are now numerous and varied. The differentiation of iPSCs into renal podocytes [77,78], hematopoietic progenitors [77], neurons [79], endothelial cells [80], cardiomyocytes [81], retinal progenitors [82], pancreatic β islet cells [83], or ciliated epithelial cells [84], implies no limits to human tissue modeling in vitro. The recent development of organoids also illustrates the progress of knowledge in the manipulation of cell fate. Three-dimensional suspension cultures of pluripotent cells allow them to organize and differentiate into spheroid structures, in which several cell types cohabit. The cells thus form “mini-organs” in which cellular interactions mimic those that exist within tissues in vivo. Organoids have become very popular in recent years [85,86,87] and many teams model tissues and characterize the cell populations in these structures with increasing precision, particularly through high-throughput single-cell transcriptomics [88]. The most advanced organoids currently model the brain [89,90,91], intestine [92,93], kidney [94], heart [91,95,96,97], or retina [98].

More recently, the emergence of cell-printing technologies, using PSCs or differentiated cells as “inks”, has also led to advances in the formation of heterogeneous tissues and has even allowed the development of supports for ear cartilage regeneration [99,100,101].

Despite the rapid advances in this field, the level of complexity attained in cellular and organoid models still falls short of the real complexity of living organisms, in which large systems interact with each other and constantly adapt to changes brought about by the environment. These modeling strategies are thus complementary approaches to animal experimentation.

#### 3.3.2. Modelling Age-Related Pathologies In Vitro

Technologies reprogramming human somatic cells into iPSCs [71,102], have also paved the way for the generation of patient-derived iPSCs, allowing the various pathological phenotypes to be recreated in vitro, e.g., for genetic disorders, including Duchenne muscular dystrophy, Becker muscular dystrophy, Parkinson’s disease, Hungtington’s disease, trisomy 21, and polymorphic catecholaminergic ventricular tachycardia [103,104,105].

Accelerated aging pathologies can also be modeled through reprogramming. Our group has modeled several of these syndromes. Indeed, we have demonstrated that cells from Werner syndrome patients can be reprogrammed while maintaining their shortened telomeres phenotype [106]. We also reprogrammed cells from a patient with Bloom syndrome, while maintaining the characteristic sister chromatid exchange phenotype [107]. Other teams have obtained similar results on several premature aging syndromes [108,109,110,111,112,113].

#### 3.3.3. New Models for the Screening of Therapeutic Molecules

In addition to providing new knowledge about the molecular characteristics of pathologies and their development, pathology models derived from hPSCs can also provide key lead molecules in high-throughput screens [114,115]. Furthermore, these screens can test potential therapeutic agents on organoids in specific pathological contexts to assess toxicity and optimize treatment.

For example, evaluating therapeutic candidates for cardiotoxicity is a major phase in drug development, and thus a particularly important application in hPSC-based models [116,117,118,119,120].

Thus, hiPSCs can be broadly used as a modelling tool. Moreover, an important parameter, brought by the use of patient-derived iPSCs, is the personalized nature of this approach, allowing hypotheses to be tested in the patient’s genetic background [121,122,123]. Furthermore, the intersection of stem cell research and genome editing research, and in particular, the recent advances in the use of CRISPR-Cas technology, promises to open up new possibilities in the correction of genetic mutations associated with pathological phenotypes [124,125,126,127]. These developments pave the way for future therapies based on cell or tissue replacement by their genetically corrected ex vivo equivalent derived from iPSCs.

## 4. New Strategies in Regenerative Medicine to Rejuvenate Cells and Tissues

**Figure 2:**
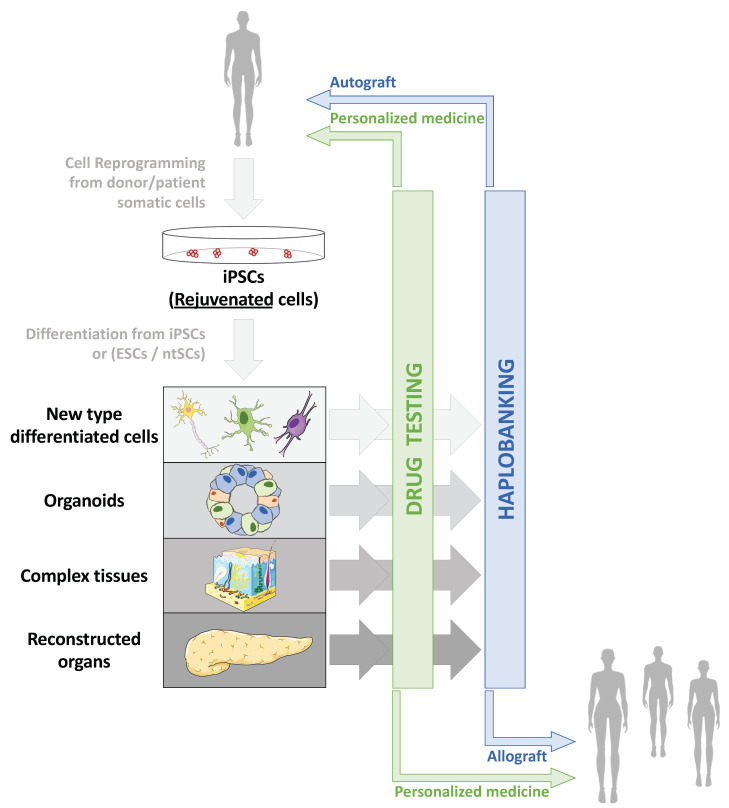
Applications of cell reprogramming and hPSCs to restore altered or aged tissues. Due to increased life expectancy and global population aging, two major health issues are arising: increased prevalence of age-associated pathologies whose mechanisms remain only partially explored and understood, and increased age-associated tissue deterioration and loss of function. Therefore, human pluripotent stem cells (hPSCs), including embryonic stem cells (ESCs), nuclear transfer stem cells (ntSCs) and induced pluripotent stem cells (iPSCs) emerged as tools to model both age-associated pathologies and tissue deterioration: from 2D cell culture to 3D complex reconstructed tissues, through organoids, and cells or tissue replacement strategies. Thanks to cell reprogramming [4,71], iPSCs made it possible to envisage autografts, especially in aged patients, as reprogramming erases aging marks in iPSCs and allows production of “rejuvenated” cells after differentiation [128].

Taking advantage of cell reprogramming, several strategies can be envisioned to rejuvenate cells and tissues. Two major types of treatment are of note. A classical therapeutic approach is the direct consequence of clinical applications based on the production of differentiated cells from iPSCs to regenerate or replace cells inside a damaged tissue or even replace the entire injured organ (Figure 2). Another more innovative and disruptive process is to act directly on the cells, inside the damaged tissue, to rejuvenate them, without modifying their identity. In the same vein, we can also imagine intervening prophylactically before the appearance of the damage induced by aging.

### 4.1. Clinical Applications of Human Pluripotent Stem Cells

All developments in the ex vivo reproduction of tissue for analytical purposes also benefit clinical applications that aimed at “repairing” humans. In contexts such as the shortage of organs to meet the demand for transplants, the inexistence of therapeutic solutions in certain cases of traumatic injuries or the problem of immune rejection of transplants after transplantation, therapies based on hPSCs and particularly iPSCs are extremely innovative and promising.

#### 4.1.1. Production of hPSCs for Clinical Use

The therapeutic use of hPSCs requires safety standards, and it is therefore highly pertinent to develop reprogramming factors that minimize the risk of alterations. For example, Okita et al. demonstrated that the transgene encoding C-MYC could be reactivated and cause tumors in chimeric mice derived from retroviral-vector-reprogrammed iPSCs [129]. Other studies have also revealed that genetic and epigenetic alterations occur during very long-term maintenance of cells in culture and that culture techniques also have an impact at this level [130,131]. Quality control of the genomic integrity of clones used for therapeutic applications should therefore be applied, even when reprogramming has been carried out using non-integrative factors [132]. There have also been refinements to the composition of hPSC culture media and matrices that ensure the absence of xenogenic elements for clinical use [132,133].

The reprogramming of patient cells, although relatively cumbersome and expensive, has tremendous advantages for autologous therapies. Cells can easily be collected by blood sampling and thus very low surgical risk is associated with very little inconvenience to the patient. Recently, culture techniques in microfluidic systems have shown an increase in the efficiency of reprogramming when mRNA-like factors are used rather than conventional culture techniques. Moreover, this approach allows a drastic reduction in the amount of components needed for reprogramming [134,135].

iPSCs can also be used for allogeneic transplants. One approach is to build haplobanks in which cells would be characterized and selected for their compatibility with the recipient, in particular for human leukocyte antigen (HLA) [136,137,138]. Another interesting possibility is to decrease cell immunogenicity, as demonstrated in mice by Deuse et al. [139]. In their experiments, they found that murine and human iPSCs lost their immunogenicity from the dual effects of CD47 overexpression and CRISPR-Cas9 ablation of major class I and II histocompatibility complexes [139]. This proof of principle suggests it will be possible to design several clones of “universal” iPSCs characterized and modified to be compatible with the general population, which would greatly reduce the cost compared to patient-specific strategies. However, such a strategy should be used with caution as it increases the risk of cancer development due to a reduction of cell immunogenicity. Therefore, in order to ensure maximum security, control of the system using suicide genes could be added [140,141].

#### 4.1.2. Cell and Tissue Replacement Therapies

Therapies based on the transplantation of cells and tissues, differentiated from hPSCs, aim to replace or repair age-related injured, damaged, or non-functional tissues [142]. We will discuss a few illustrative examples. Many cell and tissue replacement trials have focused on the nervous system and traumas, such as spinal cord injuries, that often occur in accidents. These frequently lead to reduced motor functions, even paralysis, or loss of sensory functions. Unfortunately, there are no real classical therapeutic solutions yet for these situations. Demonstrating the potential of hPSCs, it was showed, in 2005, that the transplantation of human neural stem cells of fetal origin into the spinal cord of a primate—a marmoset—can promote functional recovery after injury. In particular, it was shown that the transplanted cells differentiate into neurons, astrocytes, and oligodendrocytes [143]. The same group went on to demonstrate in mice and marmosets that human neural stem cells derived from iPSC differentiation could improve motor functions, form synaptic connections with host neurons and reduce demyelination from injury [144,145]. This cell replacement strategy was also applied for deafness using hESCs differentiated into otic progenitors and then into ciliated cells and auditory neurons. After transplantation, these cells significantly improved auditory response thresholds in a model of lesion-generated auditory neuropathy [146].

Degenerative pathologies can also benefit from this type of therapeutic approach. Neurodegenerative diseases such as Alzheimer’s and Parkinson’s are among the interesting targets for cell therapy given their frequency in the population [147,148,149]. In monkeys, autologous transplantation of dopaminergic neurons, derived from iPSCs, avoided immunosuppression and significantly re-innervated the putamen, improved motor function and enhanced survival by over two years [150]. Retinopathies, such as age-related macular degeneration or retinitis pigmentosa, have also been targeted in several clinical trials using differentiated cells derived from hESCs or iPSCs [151,152]. In 2017, Mandai et al. performed an autologous retinal cell transplant of retinal cells derived from iPSCs from a patient with neovascular (or wet) AMD [153]. Another development by Ben M’Barek et al. focusing on the treatment of retinitis pigmentosa associated with mutations in the LRAT, RPE65 and MERTK genes, used a sheet of retinal pigmentary epithelium grown on a human biological matrix of amniotic origin. This leaflet, derived from hESCs using a GMP process, has been tested in mice and primates and is currently in clinical trials [152].

#### 4.1.3. Organ Production in In Vivo Models

All the therapeutic strategies we have addressed consist of developing therapeutic cells or tissues ex vivo, under defined conditions, and then reimplanting them in the patient. Another approach consists of developing complete human organs directly in animal hosts. By creating in vivo models closer to human beings, it should be possible to generate functional and directly transplantable organs and circumvent the lack of organs [154,155].

By injecting iPSCs from one species into a blastocyst stage embryo of a second species, it is possible to generate interspecific chimeric individuals composed of cells from both species. Interspecific organogenesis then takes a specific organ of one species grown in a second species host that has a defect in the development of the organ in question. This was first performed in 2010 by Kobayashi et al., who injected rat iPSCs into a mouse blastocyst for which the genesis of the pancreas was genetically disabled by deletion of the PDX1 gene. This ‘blastocyst complementation’ resulted in a mouse with a functional mouse-sized rat pancreas [156]. The reverse experiment was performed a few years later by Yamaguchi et al., using the same genetic deletion in rats, with mouse iPSCs. Again, the host organism, the rat, had a normal rat-size functional pancreas, derived from the donor mouse cells [157]. Usui et al. showed in 2012 that it was possible to extend this process to other organs by performing intraspecific blastocyst complementation with wild-type mouse iPSCs and a mouse blastocyst, deleted for the SALL1 gene, i.e., in which kidney genesis is inactivated. The chimera resulting from the complementation also showed functional kidneys from the donor cells [158]. All these studies make it possible to envisage blastocyst complementation from hPSCs in blastocysts from animals such as pigs or sheep, whose organ size, anatomy, and physiology are close to those of human organs. However, Wu et al. have found that the frequency of human cells in chimeric pig embryos is currently very marginal [159].

Many improvements and discoveries still need to be made to make this type of strategy fully operational. Recently, it was demonstrated that the contribution of donor cells to host tissues is greatly improved by the artificial creation of a permissive niche that could even allow the formation of complete organs [160]. However, the main limitation to achieve interspecific chimerism is indisputably the pluripotent state of hPSCs. Indeed, two distinct states of pluripotency have been characterized—the naïve state corresponding to mouse ESCs and the primed state corresponding to hPSCs or to mouse epiblast stem cells (epiESCs) originating from the early post-implantation epiblast [161,162]. These different naïve and primed states have important archetypal differences, particularly in terms of cellular metabolism, the level of chromatin methylation, and gene expression. They also display important functional differences, notably in their ability to integrate into other species embryos [163]. Numerous research projects aim at developing and optimizing cell culture processes to increase the ‘naivety’ of hPSCs to approach that of murine naive cells and to increase their capacity to integrate into blastocysts [164,165,166].

### 4.2. Organismal Rejuvenation through Cellular Reprogramming

As we have just seen, the new therapeutic solutions provided by regenerative medicine benefit, or will benefit, the fight against many age-related diseases. Many age-damaged tissues and organs can already be replaced, or may be considered for replacement in the near future, thanks to ongoing innovations in stem cell research. This would be possible thanks to organs grown ex vivo or produced in animals from iPSCs derived from patient cells. However, there are obstacles to realizing this vision.

#### 4.2.1. Aging and Senescence, Two Obstacles to Reprogramming

One of these important limitations is the aging itself, of the individual, since, as we previously discussed, there are important changes that negatively and permanently affect cells as they age. Thus, developing autologous replacement strategies based on cells already altered by age would lead to the creation of new organs that are already old and therefore, by definition, damaged. Cell senescence, which is always increasing in the body during aging, is a major obstacle to cell reprogramming, reducing the effectiveness of autologous approaches in an aging context. It is notably via epigenetic remodeling of the CDKN2A locus and overexpression of the proteins p53, p16INK4A and p21CIP1 that senescence is thought to act as a barrier to reprogramming in older and damaged cells [167,168,169]. Consequently, inhibition of the p16INK4A pathway [170] or inactivation of the p53 gene [171,172] can increase reprogramming efficiency and have even enabled reprogramming in cells that failed to be reprogrammed under normal conditions, although these changes increased the susceptibility to genetic instability. The inactivation of p53 not only promoted reprogramming but also allowed reprogramming of cells via only two transcription factors: OCT4 and SOX2 [173]. One of the obstacles for reprogramming is thus falling.

Recently, Mahmoudi et al. demonstrated high variability in reprogramming in elderly fibroblast populations, due in part to the pro-inflammatory secretory profile of certain so-called “activated” fibroblasts. These fibroblasts are characterized by the secretion of inflammatory cytokines, notably TNF, and are also believed to be involved in the variability of in vivo wound healing rates in elderly mice [174].

#### 4.2.2. Cellular Reprogramming to Erase Cell Aging

In many ways, iPSCs are considered equivalent to ESCs, if not indistinguishable. Although this is still under discussion, it is clear that these cells have much in common and that iPSCs have embryonic genetic and epigenetic characteristics. Among these characteristics, some are known to be altered by age, such as telomeric shortening. Thus, by restoring an embryonic state, reprogramming has demonstrated a very interesting ability to erase certain cellular marks of aging. Marion et al. have thus shown that reprogramming fibroblasts with short telomeres resulted in an extension of the telomeres in the same way as reprogramming young fibroblasts with longer telomeres [175]. From a metabolic point of view, Surh et al. demonstrated that after reprogramming, iPSCs exhibit mitochondria similar to those of ESCs. Moreover, after redifferentiation, neo-fibroblasts significantly improved functionally, compared to their parent fibroblasts [176].

It is intuitive that re-programming promotes cell rejuvenation in certain ways, as an embryonic cell (or iPSC) has more juvenile feature than an adult cell. Furthermore, we demonstrated for the first time that cell reprogramming can even rejuvenate cells from centenarians, and that it can also overcome the barrier of cell senescence without directly inactivating senescence inducers such as p53, p16INK4A, and p21CIP1, as discussed in the previous paragraph [128]. The reprogramming protocol used has been optimized and is based on the use of a cocktail of the combined six reprogramming factors from pooling the overlapping four factor cocktails of Yamanaka [71] and Thomson [102], i.e., OCT4, SOX2, KLF4, C-MYC, NANOG, and LIN28 (OSKMNL). Following this protocol, we discovered that iPSCs reprogrammed from replicative senescing or centennial cells had restored telomere and mitochondrial functions, with a gene expression profile and a level of oxidative stress similar to hESCs. In addition, after their redifferentiation, the fibroblasts obtained had reset their proliferation capacity and had a similar transcriptomic profile to fibroblasts derived from hESCs, as well as a restored metabolism. This demonstrated conclusively that “cellular aging” is reversible. Overall, then, iPSC technology is now among the major regenerative medicine approaches for elderly patients and the one that promises the most perspectives for new therapeutic avenues.

#### 4.2.3. Complete Cellular Reprogramming Causes Teratomas

As a result of all these observations, several teams around the world, including ours, have wondered whether cell rejuvenation by reprogramming could also be applied in vivo, directly within tissues, to prevent aging deteriorations. Thus, various distinct mouse models for in vivo reprogramming have been developed to explore this hypothesis.

Abad et al. were the first to address this question [177]. They developed two different functional transgenic murine lines, named i4F-A and i4F-B, both allowing the inducible expression of the four reprogramming factors in the presence of doxycycline. A polycistronic expression cassette encoding OSKM was inserted using a lentivirus-like vector into two different genome loci: an intron of the *Neto2* gene for the i4F-A lineage and into an intron of the *PPARγ* gene for the i4F-B lineage. The expression of the OSKM cassette is controlled by a doxycycline-inducible transcriptional activator (rtTA) in the Rosa26 locus. Firstly, mice were treated with a high dose, 1mg/mL, of doxycycline in drinking water to induce OSKM, which revealed a very rapid deterioration in the health of the animals after just one week, including significant weight loss and damage to the intestine and pancreas. Other protocols were then designed to minimize these effects and maximize survival, which led to the generation of pluripotent cells in vivo, circulating in the blood, and thus validated the feasibility of direct reprogramming in animals. Unfortunately, these treatments also produced teratomas in many organs, especially the pancreas, kidneys, intestine, and adipose tissue, with an incidence of over 40%. Using another in vivo reprogramming model, Ohnishi et al. achieved results similar to Abad et al. [178], with a rapid degradation of health status due to the proliferation of undifferentiated dysplastic cells within the tissues. The authors also observed the appearance of teratomas in the kidneys, pancreas, and liver, even one week after stopping a seven-day treatment on their animals. Thus, although in vivo reprogramming has deleterious effects on health status and lifespan when carried out to completion, it is nevertheless possible to convert adult cells into embryonic cells in vivo just as in cell culture.

Based on the previously described mouse transgenic models [177], several studies have revealed a strong association between reprogramming and tissue senescence. In vivo, complete reprogramming requires senescence-associated secretory phenotype (SASP) factors, in particular IL-6 [179]. Indeed, Mosteiro et al. demonstrated the role of senescence in cell plasticity by generating teratomas in the lungs, only in the context of injury. In addition to the teratomas, this organ had high expression levels of senescence markers such as IL-6 and PAI-1. Inactivation of senescence in this tissue inhibits teratoma formation. Similar results obtained in injured muscle by Chiche et al., using the same model, highlighted the central role of Pax7^+^ muscle stem cells in the reprogramming of this tissue [180].

Overall, these examples fully illustrate the importance, for any reprogramming strategy aiming at rejuvenating organisms, of first overcoming the conditions leading to deleterious total dedifferentiation of the cells.

#### 4.2.4. Partial Cellular Reprogramming Rejuvenates Cells In Vitro and In Vivo

To overcome this ultra-dedifferentiation problem, Ocampo et al. have developed a protocol to induce partial reprogramming. Their work was the first proof that reprogramming can counteract aging, demonstrating in particular that cyclic expression of OSKM in vivo can prolong the life expectancy of mice recapitulating the human Hutchinson–Gilford Progeria Syndrome, while improving the age-related phenotype [181]. For the purposes of their experiments, the authors used reprogrammable homozygous progeria mice of genotype Lmna^G609G/G609G^ R26^rtTA/+^ Col1A1^4F2A/+^ obtained by crossing a reprogramming model developed by Carey et al. [182] with the accelerated aging model developed by Osorio et al. [183]. The authors chronically induced OSKM with a dose of 1mg/mL of doxycycline in bottle water two days per week. It was observed that by following this induction protocol, the life expectancy of homozygous progeria animals was increased by almost a third, with a median life expectancy of 24 weeks for treated animals compared to 18 weeks for controls. This improvement in longevity was also accompanied by an overall improvement in health, as well as maintenance of tissue integrity in organs such as the kidneys, spleen, stomach, and heart. These results were, however, obtained on animals with a homozygous *Lmna* gene mutation, i.e., that were highly abnormal [183]. It would be interesting to confirm these results in the context of normal physiological aging or in models closer to it, such as heterozygous progeria animals for this same mutation.

Interestingly, it was shown in the same study that induction of OSKM improves (i) the regenerative capacities of non-progeria animals of genotype Lmna^+/+^ R26^rtTA/+^ Col1A1^4F2A/+^ [181], (ii) regeneration in a model of diabetes induced by streptozocin toxin administration, and (iii) in a model of muscle degeneration induced by intramuscular cardiotoxin injection. The improvements occur through an increase in the number of Pax7^+^ satellite stem cells that are involved in muscle fiber regeneration [181]. In the same mouse model, Doeser et al. showed that local induction of reprogramming factors temporarily slowed skin wound healing by reducing the activity of fibroblasts and their transdifferentiation into myofibroblasts, illustrated by the down-regulation of the markers TGFβ1, COL1a1, and αSMA. The consequence of this phenomenon is a significant reduction in the formation of scar tissue during regeneration [184]. Recently, Rodríguez-Matellán et al. demonstrated, with the i4F-B model, that a cyclic induction three days per week by 2mg/mL of doxycycline improved cognitive functions in mice, with a positive correlation between an increase scored in object recognition memory test and the level of OSKM expression [185].

In addition, Ocampo et al. demonstrated that inducing OSKM for four days induces epigenetic rearrangement in the histone markers H3K9me3 and H4K20me3, which are known to be deregulated during aging, in vitro and in vivo in the tissues of treated animals. However, these short induction effects were reversible, suggesting that chronic induction is necessary to obtain impact on longevity [181].

To further investigate the impact of partial reprogramming in humans, Sarkar et al. recently developed an in vitro strategy based on the use of mRNA to allow the expression of the 6 OSKMNL reprogramming factors in young and old human cells [186], whose effectiveness in erasing aging hallmarks leading to a rejuvenated phenotype had previously been established by the work of our team [128]. They demonstrated, in fibroblasts and endothelial cells, that transient reprogramming could restore certain cellular characteristics altered in physiological aging, including two epigenetic clocks, namely a pan-tissue epigenetic clock based on 353 CpGs and a skin- and blood-focused second clock based on 391 CpGs, described to be highly correlated to chronological age. In addition, the authors demonstrated that reprogramming changed the level of H3K9me3, improved proteosomal activity and autophagosome formation, and decreased ROS. To analyze whether transient OSKMNL expression could also reverse age-related phenotypes such as increased levels of inflammation and decreased regenerative capacity of adult stem cells, the authors first analyzed the transcriptional consequences of reprogramming to chondrocytes in elderly osteoarthritic patients. They observed a significant reduction in intracellular mRNA levels of RANKL and iNOS2, as well as in the levels of inflammatory factors secreted by the cells, such as MIP1A, IL-6, IFNA and MCP3. In a second step, they analyzed the power and regenerative capacity of transiently reprogrammed human muscle stem cells of different ages by transplanting them into a mouse muscle injury model. Reprogrammed aged stem cells became stronger and regenerated better muscle fibers [186]. These results are promising as they open the way to new in vivo reprogramming strategies for cell therapy interventions and validate the non-integrative approach to achieve the expression of reprogramming factors.

Another type of in vivo reprogramming strategy has been illustrated recently. Senís et al. demonstrated that in vivo reprogramming was achievable by delivering OSK factors with viral vectors [187]. This kind of approach has very recently been illustrated as a strategy for regeneration of the central nervous system in mice, and more precisely, for restoring vision [188]. In this study, the authors used AAV2 vectors for the controlled expression of a polycistronic cassette encoding OSK factors that they injected into the vitreous body of the mouse eye to reach the retina. To test the safety of this strategy, the authors maintained an induction for over 15 months to validate the absence of tumors or deformations of the retina. The authors then demonstrated that the induction of OSK in retinal ganglion cells increased their survival, the regeneration of their axonal extension and forming of the optic nerve, during different stress situations. These included a model of optic nerve injury by nerve compression, a model of glaucoma induced by ocular overpressure, and their final demonstration was in the context of age-related vision impairment. DNA methylation and transcriptomic profiles were also restored in these retinal ganglion cells. Furthermore, epigenetics seems to play an important role in the regeneration phenomenon, as the inhibition of TET1 and TET2 DNA demethylase acts as a barrier and prevents any restoration. Epigenetic reorganizations involved in transient reprogramming are the widely considered to be the driving force behind the global rejuvenation phenomenon observed both in vitro and in vivo [181,185,186,188,189].

In summary, then, the various modes of cellular reprogramming detailed above confirm it as an important avenue toward innovative therapies to combat the harmful effects of aging and age-related pathologies due to decreased regenerative capacities of stem cells altered by aging.

## 5. Conclusions

**Figure 3:**
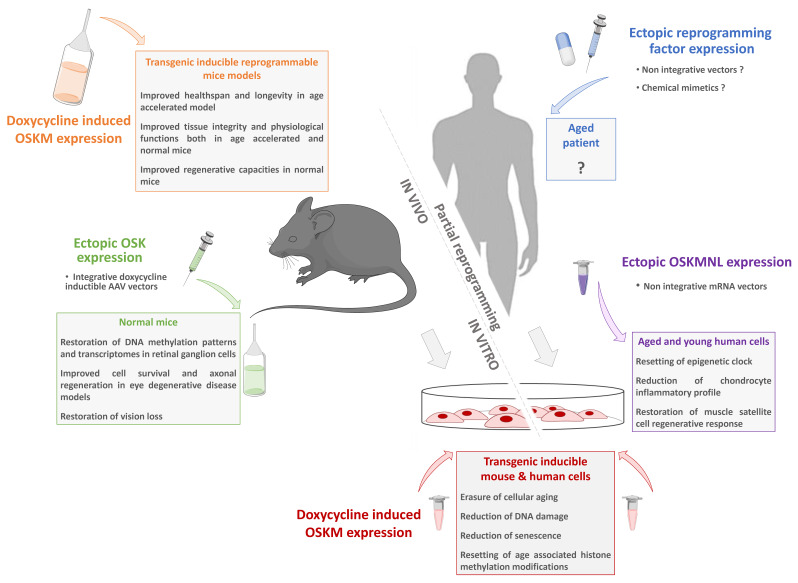
Partial reprogramming toward pluripotency as a new anti-aging strategy. For decades, complete cell reprogramming has been demonstrated to reset somatic cell physiology to a juvenile state equivalent to ESCs. Starting from transgenic models allowing inducible reprogramming factor expression to non-integrative vectors; numerous studies have recently demonstrated that a partial reprogramming is sufficient to restore the general characteristics of cellular aging without changing the identity of the cells. These innovative approaches pave the way for new strategies based on a safe transient reprogramming that can be directly transposed to humans.

The above paragraphs address the many approaches based on the properties of cell pluripotency and reprogramming that can be used to counter the multifactorial damages of aging. “Classical” approaches using iPSCs and derived cells obtained after differentiation are now being intensively studied and developed, and clinical applications, although still in their infancy, are progressing very rapidly. Beyond this, methods based on a direct intervention through partial reprogramming as a strategy against aging have laid the foundations for more disruptive approaches (Figure 3). All these procedures can be used to rejuvenate cells or tissues. Depending on the timing, the intervention can either be preventive or therapeutic. Moreover, these strategies, or a combination of them, might either delay or slow aging, or both. It is obvious that purely genetic techniques to induce reprogramming in humans are not feasible, and lifelong chronic induction is far from being translated to the clinic. However, the studies we summarize and many others that we have not had the space to cover establish a proof-of-concept for further investigations to define an optimal regimen suitable for clinical applications. Indeed, the identification of molecular and cellular pathways for tissue improvement or repair during aging opens the door for strategies for ectopic expression of reprogramming factors using non-integrative vectors or using mimetic molecules to activate endogenous reprogramming factors. In addition, these investigations could lead to the discovery of secondary and/or complementary pathways to intervene during aging and improve the healthspan. Thus, a wide range of therapeutic solutions based on induced pluripotent stem cells, but also on cell reprogramming strategies, is now available to improve healthy aging for the benefit of individuals and society.

## Data Availability

The data presented in this study are available on request from the corresponding author.
